# Effect of inulin on breath hydrogen, postprandial glycemia, gut hormone release, and appetite perception in RYGB patients: a prospective, randomized, cross-over pilot study

**DOI:** 10.1038/s41387-024-00267-5

**Published:** 2024-03-06

**Authors:** R. E. Steinert, M. Mueller, M. Serra, S. Lehner-Sigrist, G. Frost, D. Gero, P. A. Gerber, M. Bueter

**Affiliations:** 1 Department of Surgery and Transplantation, University Hospital Zurich (USZ) and University of Zurich (UZH), Zürich, Switzerland; 2 Section for Nutrition Research, Department of Metabolism, Digestion and Reproduction, Faculty of Medicine, Imperial College London, London, UK; 3 Department of Endocrinology, Diabetology and Clinical Nutrition, University Hospital Zurich (USZ) and University of Zurich (UZH), Zürich, Switzerland

**Keywords:** Obesity, Metabolic syndrome, Metabolic syndrome, Type 2 diabetes

## Abstract

**Background and objective:**

Large intestinal fermentation of dietary fiber may control meal-related glycemia and appetite via the production of short-chain fatty acids (SCFA) and the secretion of glucagon-like peptide-1 (GLP-1) and peptide YY (PYY). We investigated whether this mechanism contributes to the efficacy of the Roux-en-Y gastric bypass (RYGB) by assessing the effect of oligofructose-enriched inulin (inulin) vs. maltodextrin (MDX) on breath hydrogen (a marker of intestinal fermentation), plasma SCFAs, gut hormones, insulin and blood glucose concentrations as well as appetite in RYGB patients.

**Method:**

Eight RYGB patients were studied on two occasions before and ~8 months after surgery using a cross-over design. Each patient received 300 ml orange juice containing 25 g inulin or an equicaloric load of 15.5 g MDX after an overnight fast followed by a fixed portion snack served 3 h postprandially. Blood samples were collected over 5 h and breath hydrogen measured as well as appetite assessed using visual analog scales.

**Results:**

Surgery increased postprandial secretion of GLP-1 and PYY (*P* ≤ 0.05); lowered blood glucose and plasma insulin increments (*P* ≤ 0.05) and reduced appetite ratings in response to both inulin and MDX. The effect of inulin on breath hydrogen was accelerated after surgery with an increase that was earlier in onset (2.5 h vs. 3 h, *P* ≤ 0.05), but less pronounced in magnitude. There was, however, no effect of inulin on plasma SCFAs or plasma GLP-1 and PYY after the snack at 3 h, neither before nor after surgery. Interestingly, inulin appeared to further potentiate the early-phase glucose-lowering and second-meal (3–5 h) appetite-suppressive effect of surgery with the latter showing a strong correlation with early-phase breath hydrogen concentrations.

**Conclusion:**

RYGB surgery accelerates large intestinal fermentation of inulin, however, without measurable effects on plasma SCFAs or plasma GLP-1 and PYY. The glucose-lowering and appetite-suppressive effects of surgery appear to be potentiated with inulin.

## Introduction

The efficacy of Roux-en-Y gastric bypass (RYGB) surgery in the treatment of obesity and obesity-related health conditions such as type 2 diabetes mellitus (T2DM) suggests that the gastrointestinal (GI) tract and secretion of GI peptides controlling satiation and meal-related glycemia play a critical role in glucometabolic health [1]. To what extent the large intestinal microbiome and its metabolic products are involved in this is not yet fully understood.

Several studies report a significant effect of RYGB surgery on gut microbial composition and metabolic activity with a reduction in fecal short-chain fatty acids (SCFAs) [2–5], i.e., acetic-, propionic-, and butyric- acid which are the main metabolic outputs of colonic fermentation. SCFAs are thought to contribute to many of the beneficial effects that have been associated with a healthy and diverse gut microbiome [6]. For example, SCFAs stimulate the secretion of GLP-1 and PYY and, thus, may activate eating-inhibitory and glucose-lowering mechanisms exerting glucometabolic health benefits. This has been suggested to underpin the effects seen after RYGB surgery on glycemia and appetite, although the reduction in fecal SCFAs as observed postoperatively in many studies [2–5] seems to contradict such a mode of action. It requires consideration, however, that the majority of SCFAs is absorbed within the colon and only a minor proportion (5–10%) is excreted in feces [7]. Moreover, in humans with obesity, SCFAs are significantly increased in feces [8] while in genetically obese mice there was an increased capacity to extract SCFAs from the diet providing additional energy to the host (about 10% of total energy from the diet) as compared to lean littermates [9].

Under physiological conditions in humans, a meal takes about 3–6 h or longer (depending on meal composition and inter-individual variability of intestinal transit) until it reaches the large intestine to be fermented and metabolized to SCFAs. Thus, the involvement of SCFAs in the regulation of acute, meal-related appetite and glycemia in healthy subjects has been questioned. Rather, the so-called “second-meal effect” has been described for fermentable fibers to modulate glycemic responses at later meals on the same or even subsequent days due to colonic fermentation and increased production of SCFAs [10, 11].

We hypothesized that the anatomical modifications after the RYGB result in an accelerated transit of fermentable fiber to the large intestine and, therefore, a more rapid and more pronounced fermentation and production of SCFAs which enhances the secretion of GLP-1 and PYY with beneficial effects on meal-related glycemia and appetite. To test this hypothesis, we performed a randomized prospective pilot study in RYGB patients before and 8 months after surgery to investigate the effect of inulin vs. maltodextrin (MDX) on breath hydrogen (as a marker of colonic fermentation), plasma SCFAs, GI peptide secretion and meal-related glycemia and appetite perception.

## Methods

### Study design

**Fig. 1:**
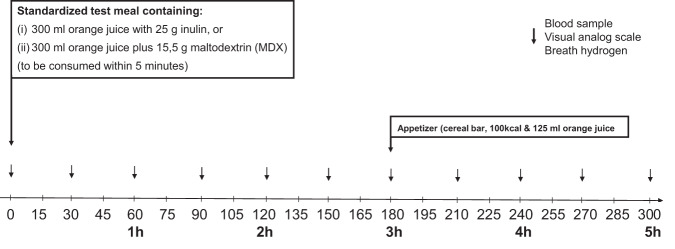
Schematic representation of the study design. Patients reported to the laboratory before and 6–8 months after the operation to receive in random order, on separate days, after fasting overnight for at least 10 h, 300 ml of orange juice (including 27 g of carbohydrate sugar) supplemented with either 25 g oligofructose-enriched inulin or an equicaloric load of 15.5 g maltodextrin (MDX). Patients had to consume the test meal within 5 min. Blood samples were taken before the test meal and postprandially at regular time intervals and breath hydrogen was collected and a visual analog scale (VAS) questionnaire was filled in to assess appetite perceptions. At *t* = 180 min, the subject consumed a fixed portion snack.

The study was designed as an explorative pilot study, following a prospective, cross-over design including eight patients (1 male, 7 female) with obesity undergoing RYGB surgery. Patients reported to the laboratory before the operation and 6–8 months after the operation to receive in random order, on separate days, after fasting overnight for at least 10 h, 300 ml of orange juice (containing 27 g of carbohydrate sugar) supplemented with either 25 g oligofructose-enriched inulin (inulin, Orafti®Synergy1, ~92% inulin content, BENEO GmbH, Mannheim, Germany), or an equicaloric load of 15.5 g maltodextrin (MDX, Nutricia GmbH, Erlangen, Germany, see Supplemental Table 1). Patients had to consume the test meal within 5 min. Blood samples were taken before the test meal and postprandially at regular time intervals over 300 min to measure blood glucose, plasma insulin, GLP-1, and PYY levels. In addition, breath hydrogen (as an indicator of large intestinal fermentation) was collected and a visual analog scale (VAS) questionnaire filled in to assess appetite perceptions (Fig. 1). At *t* = 180 min, subject consumed a fixed portion snack (cereal bar plus 125 ml orange juice (including 12 g of carbohydrate sugar); total kcal about 150 kcal) to stimulate the gastrocolic reflex facilitating large intestinal fermentation of fiber; similar experimental paradigms have been used previously [12, 13].

### Patients

The study was carried out according to the Declaration of Helsinki. Ethical approval was received from the Cantonal Ethical Committee of Zurich (BASEC-Nr. 2017-02287) and the protocol was registered at ClinicalTrials.gov with Identifier NCT03573258. All patients were informed in detail about the risks and benefits of the surgery and test meals and each provided written, informed consent to participate in the trial. Computer-generated random numbers were used to assign the order of test meals. All operations were performed laparoscopically and by the same surgical team. The RYGB technique consisted of a small gastric pouch with a linearly stapled gastrojejunostomy (30 mm) along with a 150 cm antecolic alimentary limb and a 70 cm of biliopancreatic limb. Table 1 provides subjects BMI and fasting blood glucose and plasma insulin before and after surgery.

**Table 1:** Subject BMI and fasting blood glucose and plasma insulin.

Parameter	Preoperatively^a^	Postoperatively^a^ 8 months (5-13)	*P*-value^b^
BMI (kg/m^2^)	41.5 ± 3.3	29.4 ± 4.2	<0.001
Fasting glucose (mmol/l)	5.8 ± 0.7	5.1 ± 0.4	0.046
Fasting insulin (mU/ml)	25.4 ± 9.5	8.0 ± 7.1	0.005

^a^Mean ± SD.

^b^The *P*-value was calculated based on the test statistic *W* of a Wilcoxon signed-rank test.

### Measurements

#### Blood glucose and plasma hormones

Blood samples were collected into ice-chilled EDTA tubes and plasma was separated by centrifugation at 3200 rpm for 15 min at 4 °C within 15 min of collection and stored at –80 °C until assayed. Plasma glucose was measured by a glucose oxidase method (Rothen Medizinische Laboratorien AG, Basel, Switzerland; range of assay, 0.6 to 45.0 mmol/l). Plasma insulin was quantified using a chemiluminescent microparticle immunoassay (Rothen Medizinische Laboratorien AG, Basel, Switzerland, 0.4–1000 μU/ml). Total plasma glucagon-like peptide-1 (GLP-1) and peptide YY (PYY) were measured with Sigma-Aldrich human ELISA kits (EZGLP1T-36K and EZHPYYT66K).

#### Breath hydrogen

Breath hydrogen was measured using an ambulatory hydrogen breath test (HydroCheck; Neomed GmbH, Korschenbroich, Germany).

#### Plasma SCFA

Plasma was extracted and analyzed using ultra-high-performance liquid chromatography with tandem mass spectrometry (UHPLC-MS/MS) as previously described in detail. The limit of quantitation (LOQ) for all SCCAs was at 3.3 times the Lowest limit of detection (LLOD) (s/n) ≥33 following recommended values for chromatographic validation of metabolites [14].

#### Appetite perception

Perceptions of satiety, hunger, fullness, desire to eat, prospective consumption, and nausea were quantified using validated 100-mm VAS questionnaires [15].

#### Viscosity of the test solutions

Rheological characterizations of the test solutions were performed on a strain-controlled oscillatory shear rheometer (MCR 502; Anton Paar) equipped with a Peltier stage allowing to measure viscosity at 4, 25, and 37 °C. Measurements (10 repetitions) were acquired with a 19-mm parallel plate geometry with sandblasted surface at a 1-mm measuring gap. Shear rates of 50–1000 1/s were applied to measure shear stress and viscosity in the low-viscosity mode of the rheometer. The most stable measurements were achieved at shear rates between 100 and 200 1/s and showed that while viscosities of both solutions were comparable at 4 °C and 25 °C, orange juice supplemented with inulin had a significantly higher viscosity at 37 °C than orange juice supplemented with MDX (Supplemental Fig. 1)

### Statistical analysis

No power analysis was possible due to an unknown effect size of RYGB surgery on large intestinal fermentation of inulin (assessed via breath hydrogen concentrations). Thus, the group size was chosen based on practical considerations and previous studies in our laboratory with RYGB patients [16–18]. A preliminary analysis of the data was performed to identify (1) significant outliers (box plot method) and to test (2) normality (Shapiro-Wilk test), and (3) homoscedasticity (homogeneity of variance, Levene’s test) of all measured variables.

To account for the unbalanced nature of the data driven by the small sample size (*n* = 8), and for missing values, a linear mixed model (LMM) was fitted to the data by using the maximum likelihood estimation (MLE) method to study the interaction of the test conditions (MDX preoperative; inulin preoperative; MDX postoperative; and inulin postoperative) with the 11 postprandial time points. The LMMs were fitted separately for test conditions with inulin and test conditions with MDX. The models were fitted with two within-subject predictors (test condition and postprandial time points) for two repeated observations clustered within subjects. Random intercepts were added to the model to account for the correlation of the observations within the study participants. Two nested models, with and without interaction of the fixed effects (test conditions and postprandial time points), were fitted to the data. The models were compared with Pearson’s chi-squared test (*χ*^2^) to evaluate how likely it was that any observed difference between the two models arose by chance (*H*_0_). The alternative hypothesis (H_1_) considered the difference between the two models to be driven by the interaction of the fixed effects. According to the *χ*^2^ either the model without or with interaction, respectively, was retained for further analysis. Estimated marginal means (EMMs) were estimated in the fitted LMM. Contrasts were generated for pairwise comparisons of the four test conditions (MDX preoperative against MDX postoperative; and inulin preoperative against inulin postoperative) at each postprandial time point and for each postprandial time in relation to the baseline (fasting) value. An interaction plot of EMMs was created based on the fitted LMM. The significance of the postoperative differences from the baseline values of BMI, fasting glucose, and fasting insulin was assessed with a Wilcoxon signed-rank test. The significance value was set at 0.05. All *P*-values of the pairwise comparisons were adjusted with a Bonferroni correction to account for multiple testing.

Additionally, we investigated the relationship between desire-to-eat ratings and breath hydrogen levels by calculating the area under the curve (AUC) for both measures at specific postprandial intervals using the trapezoidal rule. The correlation analysis was performed defining the AUC of either early-phase breath hydrogen (60–180 min after the start of the test) or second-meal breath hydrogen (210–300 min after the start of the test) as the independent variable and the AUC of second-meal desire-to-eat ratings (210–300 min after the start of the test) as the dependent variable. A Pearson’s correlation coefficient was computed to assess the linear relationship between the variables.

Data analysis was performed using R (version 4.0.3) (R Core Team, 2020) via RStudio (version 1.3.1093) (RStudio Team, 2020). The emmeans R package (version 1.7.1-7) was used for the estimation of EMMs and contrasts. All figures were organized using Adobe Illustrator (version 25.0.1).

## Results

### Body weight and fasting glycemia

There was a significant reduction in BMI, fasting plasma insulin, and blood glucose concentrations eight months after RYGB surgery when compared to preoperative values (*P* ≤ 0.05, respectively, Table 1).

### Blood glucose and plasma hormones

#### Blood glucose

**Fig. 2:**
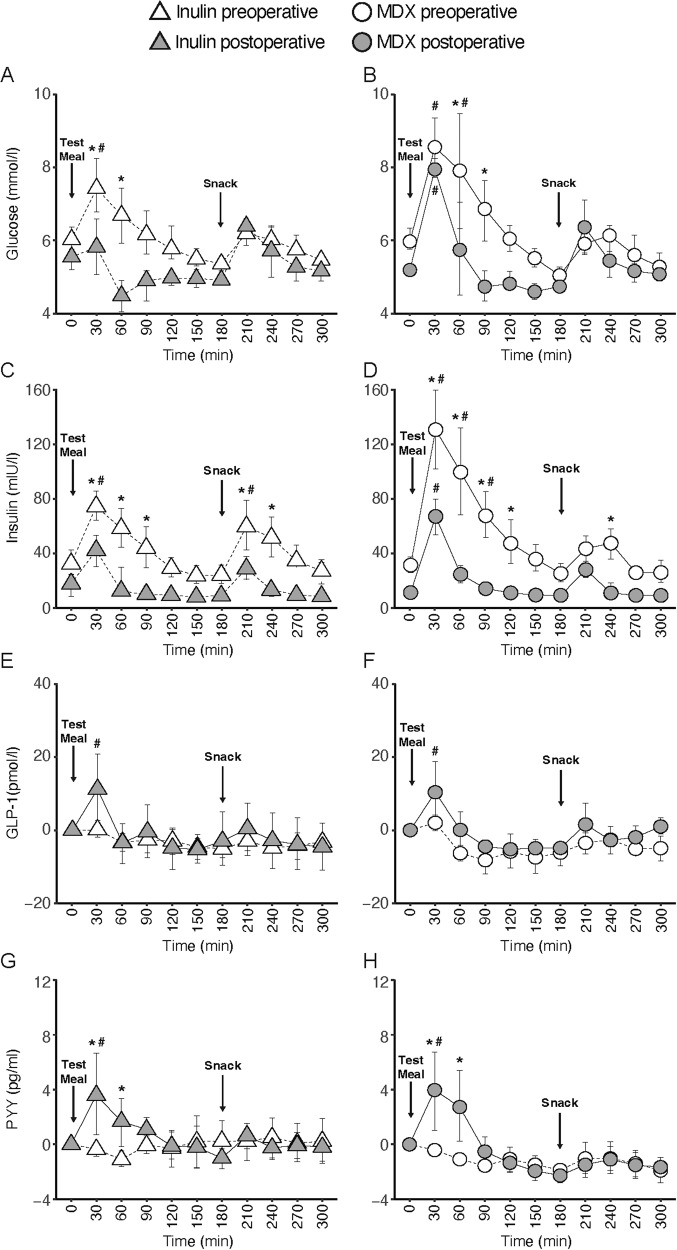
Effects of inulin and maltodextrin on postprandial glycemia and gut hormone release. Blood glucose (**A**, **B**), insulin (**C**, **D**), total GLP-1 (**E**, **F**), and total PYY (**G**, **H**) concentrations following a test meal with inulin or maltodextrin (MDX) before and after RYGB surgery. At *t* = 180 min, the subject consumed a fixed portion snack. Data are expressed as means ± SD; *n* = 8. To assess differences between treatments, the following pairwise comparisons were performed **A** effect of surgery with inulin (i.e., inulin preop vs. inulin postop; **B** effect of surgery with MDX (i.e., MDX preop vs. MDX postop) and **C** within-group comparisons from baseline (*t* = 0 min) for each of the four test conditions. ^#^*P* ≤ 0.05 vs. baseline; ^*^P ≤ 0.05 vs. preop.

The effects of inulin and MDX were comparable before surgery. Both treatments significantly increased blood glucose concentrations when compared to baseline (inulin at 30 min and MDX at 30 and 60 min; *P* ≤ 0.05, respectively). After surgery, we observed a significant reduction in postprandial blood glucose increments in response to inulin at 30 and 60 min and in response to MDX at 60 and 90 min (*P* ≤ 0.05 vs. before surgery, respectively). The glucose-lowering effect of inulin after surgery appeared to be stronger than that of MDX given that after surgery there was no significant increase from baseline with inulin while this was still the case with MDX at 30 min (Fig. 2A, B). An interaction term for the fixed effects provided a better model of the variance of the data of both inulin and MDX (*P* < 0.001) with conditional *R*^2^ of 69% and 72%, respectively.

#### Plasma insulin

Before surgery, both treatments significantly increased plasma insulin concentrations when compared to baseline (inulin at 30 min and after the snack at 210 min and MDX between 30 and 90 min; *P* ≤ 0.05, respectively). After surgery, there was a significant reduction in plasma insulin increments in response to inulin between 30 and 90 min and after the snack between 210 and 240 min, and in response to MDX between 30 and 120 min and at 240 min (*P* ≤ 0.05 vs. before surgery, respectively, Fig. 2C, D). An interaction term for the fixed effects provided a better model of the variance of the data of both inulin and MDX (*P* < 0.001) with conditional *R*^2^ of 85% and 80%, respectively.

#### Plasma total GLP-1 and PYY

Before surgery, there was no effect of inulin or MDX on plasma GLP-1 or PYY concentrations. After surgery, plasma GLP-1 and PYY concentrations increased significantly at 30 min postprandially in response to both inulin and MDX when compared to baseline (*P* ≤ 0.05, respectively, Fig. 2E, H). In addition, plasma PYY concentrations were significantly higher at 30 and 60 min when compared with before surgery in response to both inulin or MDX (*P* ≤ 0.05, respectively, Fig. 2G, H). There was, however, no increase in plasma GLP-1 or PYY concentrations after the snack at 180 min, neither before nor after surgery. An interaction term for the fixed effects provided a better model of the variance of the data of both inulin and MDX (*P* < 0.001) with a conditional *R*^2^ of 74% for GLP-1 and 67% for PYY.

### Breath hydrogen and plasma SCFA

#### Breath hydrogen

**Fig. 3:**
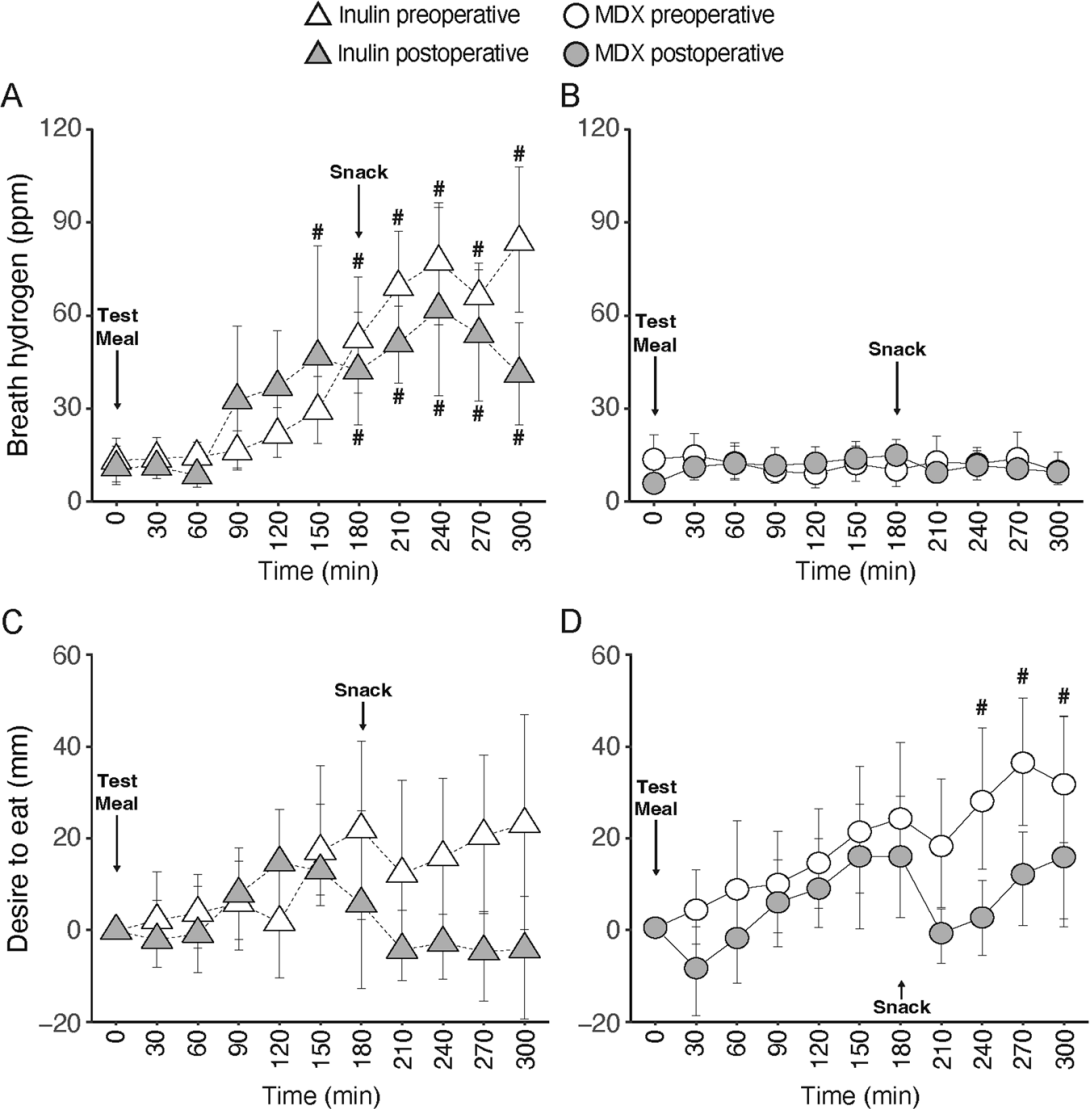
Effects of inulin and maltodextrin on breath hydrogen and appetite perception. Breath hydrogen concentrations (**A**, **B**) and scores for “desire to eat” (**C**, **D**) following a test meal with inulin or maltodextrin (MDX) before and after RYGB surgery. At *t* = 180 min, the subject consumed a fixed portion snack. Data are expressed as means ± SD; *n* = 8. To assess differences between treatments, the following pairwise comparisons were performed **A** effect of surgery with inulin (i.e., inulin preop vs. inulin postop; **B** effect of surgery with MDX (i.e., MDX preop vs. MDX postop) and **C** within-group comparisons from baseline (*t* = 0 min) for each of the four test conditions. ^#^*P* ≤ 0.05 vs. baseline; ^*^P ≤ 0.05 vs. preop.

There was no effect of MDX on breath hydrogen concentrations before or after surgery. In contrast, inulin significantly increased breath hydrogen concentrations before surgery between 180 and 300 min when compared to baseline (*P* ≤ 0.05). After surgery, this increase was earlier in onset but less pronounced in magnitude with a significant increase between 150 and 300 min when compared with baseline (*P* ≤ 0.05, Fig. 3A, B). An interaction term for the fixed effects provided a better model of the variance of the data only for inulin (*P* < 0.01). The conditional R^2^ was 67% for the model of inulin and 63% for the model of MDX.

#### Plasma SCFAs

Lactate, acetate, 2-hydroxybutyrate, and isovalerate were detected in plasma samples of most subjects but we failed to detect any effect of inulin, MDX, or surgery on plasma SCFAs. Propionate, butyrate, isobutyrate, 2-methylbutyrate, valerate, and hexanoate were either not detected or detected only in plasma samples of some subjects (data not shown). An interaction term for the fixed effects did not provide a better model of the variance of the data neither for inulin nor for MDX (*P* < 0.01). The marginal *R*^2^ was 31% and 28%, respectively.

### Appetite perception

The effect of inulin and MDX on “desire to eat” ratings was comparable before surgery, although ratings significantly increased between 240 and 300 min when compared with baseline (*P* ≤ 0.05) only with MDX but not with inulin. After surgery, we observed a reduction in “desire to eat” ratings with both, inulin and MDX. The appetite-lowering effect appeared to be more pronounced in response to inulin after the snack at 180 min given postprandial ratings fell below baseline which was not the case with MDX. All other appetite ratings followed similar patterns, however, with no significant effects between treatment or when compared with baseline (Fig. 3C, D). An interaction term for the fixed effects provided a better model of the variance of the data of both inulin (*P* < 0.001) and MDX (*P* < 0.01). The conditional *R*^2^ was 70% for the model of inulin and 66% for the model of MDX.

### Correlation of “desire to eat” ratings and breath hydrogen

**Fig. 4:**
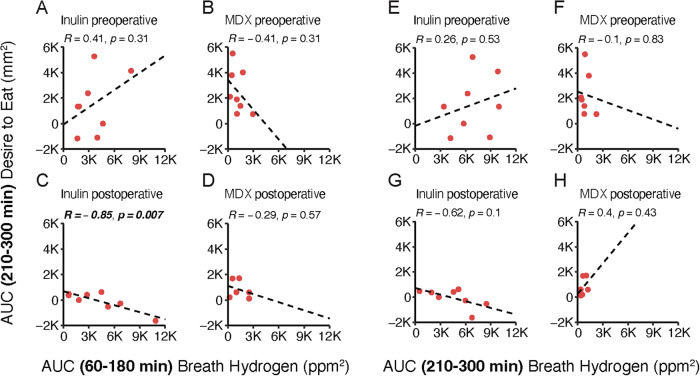
Associations between breath hydrogen and appetite perception. Correlation analysis of linear relationships between area under the curves (AUCs) for second-meal “desire to eat” ratings (210–300 min) and either (**A**–**D**) early-phase (60–180 min) as well as (**E**–**H**) second-meal (210–300 min) breath hydrogen concentrations before and after surgery. *p,* *p*-value of the coefficient of determination, *R*^2^, coefficient of determination of the linear relationship.

After surgery, the AUC for second-meal “desire to eat” ratings (210–300 min) inversely correlated with the AUC for early-phase breath hydrogen concentrations (60–180 min) in response to inulin (Fig. 4A–H, *R* = 0.85, *p* = 0.007). No other significant correlations were observed in different phases or with other parameters.

## Discussion

We investigated whether the anatomical modifications following RYGB surgery result in an accelerated transit of inulin to the large intestine and, therefore, a more rapid and pronounced fermentation and production of SCFAs with enhanced secretion of GLP-1 and PYY and beneficial effects on meal-related glycemia and appetite. This was compared to a standard, equicaloric load of MDX. Consistent with previous studies [1, 17, 19, 20], we found that surgery increased postprandial secretion of GLP-1 and PYY; lowered postprandial blood glucose and plasma insulin increments, and reduced postprandial appetite ratings in response to both inulin and MDX. While there was no effect of MDX on breath hydrogen concentrations, the effect of inulin on breath hydrogen was accelerated after surgery with an increase that was earlier in onset (2.5 h vs. 3 h) but less pronounced in magnitude. There was no effect of inulin on plasma SCFAs, nor was there an increase in plasma GLP-1 or PYY concentrations after the snack at 3 h, neither before, nor after surgery. Interestingly, inulin appeared to further potentiate both, the early-phase glucose-lowering and second-meal (3–5 h) appetite-suppressive effects of surgery when compared with MDX. Moreover, there was a strong inverse correlation between early-phase breath hydrogen concentrations (60–180 min) and second-meal “desire to eat” ratings (210–300 min) suggesting a specific and significant physiological interaction with accelerated fermentation of inulin leading to improved appetite-suppression after RYGB surgery

One obvious explanation for inulin to further potentiate the early-phase glucose-lowering effect of surgery in the absence of marked differences in early hormonal responses (GLP-1 and PYY) between inulin and MDX is likely related to the increase in orange juice viscosity at 37 °C body temperature after ingestion (see Supplemental Fig. 1). This may have slowed glucose absorption and thereby secretion of insulin from pancreatic beta cells by increasing the thickness of the unstirred water layer. That the effect was evident only in the first 90 min postprandially but not after the fixed portion snack at 3 h further argues for such an acute mechanism independent of second-meal effects that we had hypothesized. Similar effects of highly viscous fibers (e.g., oat and barley beta-glucans) on meal-related glycemia have been documented before in healthy subjects [21, 22]. The primary mechanism of action has been speculated to involve their ability to increase the viscosity of contents of the upper GI tract and, hence, slow gastric emptying [23]. The rate of gastric emptying is well established to have a substantial impact on postprandial glycemia by determining glucose absorption and incretin hormone secretion [24, 25]. In RYGB patients, however, a slowing of gastric emptying is unlikely to play a significant role given the lack of pyloric control of emptying [18]; moreover, only a fraction of the normal gastric volume can be accommodated due to the greatly reduced gastric lumen. In fact, it has been speculated that the anatomical modifications may result in an almost immediate appearance of particularly liquid meals within 1–2 min in the alimentary limb [1]. Thus, in the absence of proper control of gastric emptying after surgery, chyme viscosity may be a key determinant of postprandial blood glucose and insulin increments. Additional studies with viscous fibers such as grain beta-glucans or pectins are clearly warranted to better understand whether this can be a useful addition to diets in post-bariatric patients.

Several studies further suggest the relevance of the fermentation of prebiotic fibers and the production of SCFAs in glycaemic control and appetite management [13, 26–31]. A so-called “second-meal effect” has been described for fermentable fibers to modulate glycaemic responses and appetite not only at the first subsequent meal after consumption but also at later meals on the same or even subsequent days due to colonic fermentation and increased production of SCFA [10, 11]. For example, Cani et al. [27]. demonstrated in normal-weight subjects that 16 g inulin per day consumed over 2 weeks increased breath hydrogen excretion and plasma GLP-1 and PYY concentrations while it lowered hunger rates and postprandial plasma glucose responses after a standardized meal. The molecular mechanism linking SCFA production to the secretion of gut hormones and the control of appetite and glycemia is thought via the activation of G-protein coupled receptors including GPR41 and GPR43 on enteroendocrine cells [30].

Under acute conditions, Wolever and co-workers investigated the effects of inulin and resistant starch on postprandial SCFAs, and gut hormone responses in healthy subjects with overweight and obesity vs. lean subjects [12, 13], an experimental setup which is closer to the protocol used in our study. Overnight-fasted participants consumed 75 g glucose as control or 75 g glucose plus 24 g inulin, or 28.2 g resistant starch, and blood was collected at intervals over 6 h. A standard lunch was served 4 h after the test drink. The authors found that relative to glucose, inulin, but not resistant starch, significantly increased SCFAs from 4–6 h postprandially while neither inulin nor resistant starch affected GLP-1 or PYY. There was no effect of inulin on second-meal glucose and insulin responses in contrast to resistant starch that lowered second-meal glucose and insulin responses at 4–6 h.

Our data are consistent with these findings, we also failed to observe significant effects of inulin on GLP-1 and PYY release or second-meal glycemia following the snack at 3 h. It suggests that prolonged administration with longer adaptation periods to increase colonic fermentation (but not single interventions) may be required for these effects in RYGB patients. We are not aware of any other acute studies that have investigated second-meal effects of prebiotic fibers in patients after RYGB, however, in a recent study in 32 RYGB patients supplemented with probiotic only or probiotic plus inulin for 6 months, patients showed an increase in fasting and postprandial GLP-1 and PYY levels [32].

The early-phase hormonal responses after surgery were comparable between inulin and MDX; both resulted in significant increases in plasma GLP-1 and PYY concentrations at 30 min postprandially when compared with baseline. The relatively moderate increases after surgery are likely related to the small glucose load. It is known that oral protein and lipids typically produce more sustained increases compared to glucose which usually results in monophasic increases [1]. Moreover, the early increase in plasma GLP-1 and PYY with inulin seems controversial given that inulin is not a digestible carbohydrate. However, inulin was administered with orange juice containing 27 g of carbohydrate sugars. Moreover, whether inulin may have directly stimulated some early-phase GLP-1 release independent of its colonic fermentation to SCFAs is unknown. Wölnerhanssen et al. reported that calorie-free polyols such as erythritol and xylitol increase CCK and GLP-1 secretion [33], suggesting that a similar mechanism may account for oligofructose-enriched inulin as used in this study.

With regard to postprandial plasma SCFAs, Wolever and colleagues reported consistent increases in plasma butyrate, propionate, and acetate in response to 24 g of inulin (but not resistant starch) within a 6 h interval in healthy subjects [12]. In our study, we failed to detect increases in plasma lactate, acetate, 2-hydroxybutyrate, and isovalerate, moreover, other organic acids including propionate, and butyrate were not detected consistently despite a comparable dose. Lack of accurate quantification might be one reason for this negative finding although we used a method that was recently validated as a selective and robust LC-MS/MS-based approach to quantitate short-chain carboxylic acids in different human biofluids [14]. However, because only a fraction of colonic-produced SCFAs reach the systemic circulation due to extensive metabolism in colonocytes and liver, SCFA remains difficult to analyze in plasma with even the choice of blood tube that can affect the results. In our study we used EDTA tubes which are not ideal because they can induce acetate contamination [34, 35], however, the collection was consistent across the cohort.

Despite that we failed to detect an increase in plasma SCFAs or plasma GLP-1 and PYY in response to inulin after surgery, inulin appeared to further potentiate the effect of surgery on second-meal (3–5 h) appetite responses, particularly “desire to eat” ratings. Although these differences did not reach statistical significance, they suggest the use of prebiotic fibers to improve post-bariatric dietary management. The underpinning mechanisms, however, require further research. That second-meal “desire to eat” ratings (210–300 min) were inversely correlated with early-phase breath hydrogen concentrations (60–180 min) in the absence of any other significant correlations suggests that accelerated fermentation of inulin may have triggered additional appetite-suppressive effects. However, we failed to detect an effect on plasma GLP-1 and PYY, although other mechanisms may have been involved such as changes in postprandial ghrelin, CCK or leptin secretion, or hepatic metabolism of SCFA, so-called “energostatic” signaling [30, 36]. Also, local increases in active forms of GLP-1 in the splanchnic circulation and subsequent receptor activation may have been involved which we were unable to detect in plasma given that only 10-15% of GLP-1 is estimated to enter the systemic circulation [37].

There are a number of limitations that require consideration when interpreting our findings. First, we only included eight subjects (7 female, 1 male) in this explorative pilot study which limits the full interpretation of the data. Future studies with bigger group sizes may detect fine differences in the dynamics of breath hydrogen, gut hormone secretion, or plasma SCFA. Also, separate studies in both men and women may be warranted given the disparities in glycemic and incretin responses between sexes that have recently been reported [38]. Second, we took plasma samples only up to 5 h postprandially, however, to comprehensively investigate second-meal effects, sampling for longer periods and/or longer-term supplementation of fiber to increase colonic fermentation may be required. Finally, additional measurements of plasma ghrelin or CCK might provide further insights into the underpinning mechanism.

Taken together, we found that RYGB surgery accelerated large intestinal fermentation of inulin indicating faster delivery of inulin to the large intestine. While surgery increased postprandial secretion of GLP-1 and PYY; lowered postprandial blood glucose and plasma insulin increments and reduced postprandial appetite ratings in response to both inulin and MDX, we failed to detect an effect on plasma SCFAs. Moreover, there was no second-meal increase in plasma GLP-1 or PYY concentrations after the snack at 3 h, neither before, nor after surgery. Interestingly, inulin appeared to further potentiate both, the early-phase glucose-lowering and second-meal appetite-suppressive effects of surgery with the latter showing a strong correlation with early-phase breath hydrogen concentrations. This suggests that accelerated fermentation of inulin after RYGB surgery may trigger additional appetite-suppressive effects and accordingly that prebiotic fibers should be further investigated in longer-term studies for its potential addition to diets in post-bariatric patients

### Supplementary information

Supplemental Material

## Data Availability

The datasets generated and analyzed during the current study are available from the corresponding authors upon reasonable request.

## References

[1] Steinert RE, Feinle-Bisset C, Asarian L, Horowitz M, Beglinger C, Geary N (2017). Ghrelin, CCK, GLP-1, and PYY(3–36): secretory controls and physiological roles in eating and glycemia in health, obesity, and after RYGB. Physiol Rev.

[2] Farup PG, Valeur J. Changes in faecal short-chain fatty acids after weight-loss interventions in subjects with morbid obesity. Nutrients. 2020; 12. 10.3390/NU12030802. 10.3390/nu12030802 PMC7146446 32197409

[3] Tremaroli V, Karlsson F, Werling M, Ståhlman M, Kovatcheva-Datchary P, Olbers T (2015). Roux-en-Y gastric bypass and vertical banded gastroplasty induce long-term changes on the human gut microbiome contributing to fat mass regulation. Cell Metab.

[4] Coimbra VOR, Crovesy L, Ribeiro-Alves M, Faller ALK, Mattos F, Rosado EL. Gut microbiota profile in adults undergoing bariatric surgery: a systematic review. Nutrients. 2022; 14. 10.3390/NU14234979. 10.3390/nu14234979 PMC9738914 36501007

[5] Meijer JL, Roderka MN, Chinburg EL, Renier TJ, McClure AC, Rothstein RI (2022). Alterations in fecal short-chain fatty acids after bariatric surgery: relationship with dietary intake and weight loss. Nutrients.

[6] Koh A, De Vadder F, Kovatcheva-Datchary P, Bäckhed F (2016). From dietary fiber to host physiology: short-chain fatty acids as key bacterial metabolites. Cell.

[7] McNeil NI, Cummings JH, James WPT (1978). Short chain fatty acid absorption by the human large intestine. Gut.

[8] Kim KN, Yao Y, Ju SY. Short chain fatty acids and fecal microbiota abundance in humans with obesity: a systematic review and meta-analysis. Nutrients. 2019; 11. 10.3390/NU11102512. 10.3390/nu11102512 PMC6835694 31635264

[9] Turnbaugh PJ, Ley RE, Mahowald MA, Magrini V, Mardis ER, Gordon JI (2006). An obesity-associated gut microbiome with increased capacity for energy harvest. Nature.

[10] Brighenti F, Benini L, Del Rio D, Casiraghi C, Pellegrini N, Scazzina F (2006). Colonic fermentation of indigestible carbohydrates contributes to the second-meal effect. Am J Clin Nutr.

[11] Nilsson AC, Östman EM, Granfeldt Y, Björck IME (2008). Effect of cereal test breakfasts differing in glycemic index and content of indigestible carbohydrates on daylong glucose tolerance in healthy subjects. Am J Clin Nutr.

[12] Rahat-Rozenbloom S, Fernandes J, Cheng J, Gloor GB, Wolever TMS (2017). The acute effects of inulin and resistant starch on postprandial serum short-chain fatty acids and second-meal glycemic response in lean and overweight humans. Eur J Clin Nutr.

[13] Rahat-Rozenbloom S, Fernandes J, Cheng J, Wolever TMS Acute increases in serum colonic short-chain fatty acids elicited by inulin do not increase GLP-1 or PYY responses but may reduce ghrelin in lean and overweight humans. Eur J Clin Nutr. 2016. 10.1038/ejcn.2016.249. 10.1038/ejcn.2016.249 PMC5423780 27966574

[14] Valdivia-Garcia MA, Chappell KE, Camuzeaux S, Olmo-García L, van der Sluis VH, Radhakrishnan ST et al. Improved quantitation of short-chain carboxylic acids in human biofluids using 3-nitrophenylhydrazine derivatization and liquid chromatography with tandem mass spectrometry (LC-MS/MS). J Pharm Biomed Anal 2022; 221. 10.1016/J.JPBA.2022.115060. 10.1016/j.jpba.2022.115060 36166933

[15] Parker BA, Sturm K, MacIntosh CG, Feinle C, Horowitz M, Chapman IM (2004). Relation between food intake and visual analogue scale ratings of appetite and other sensations in healthy older and young subjects. Eur J Clin Nutr.

[16] Steinert RE, Rehman A, Souto Lima EJ, Agamennone V, Schuren FHJ, Gero D (2020). Roux-en-Y gastric bypass surgery changes fungal and bacterial microbiota in morbidly obese patients-A pilot study. PLoS ONE.

[17] Peterli R, Steinert RE, Woelnerhanssen B, Peters T, Christoffel-Courtin C, Gass M (2012). Metabolic and hormonal changes after laparoscopic Roux-en-Y gastric bypass and sleeve gastrectomy: a randomized, prospective trial. Obes Surg.

[18] Gero D, Steinert RE, Hosa H, Cummings DE, Bueter M. Appetite, glycemia, and entero-insular hormone responses differ between oral, gastric-remnant, and duodenal administration of a mixed-meal test after roux-en-y gastric bypass. Diabetes Care 2018; 41. 10.2337/dc17-2515. 10.2337/dc17-2515 29636353

[19] le Roux CW, Aylwin SJB, Batterham RL, Borg CM, Coyle F (2006). Gut hormone profiles following bariatric surgery favor an anorectic state, facilitate weight loss, and improve metabolic parameters. Ann Surg.

[20] Borg CM, Le Roux CW, Ghatei MA, Bloom SR, Patel AG, Aylwin SJB (2006). Progressive rise in gut hormone levels after Roux-en-Y gastric bypass suggests gut adaptation and explains altered satiety. Br J Surg.

[21] Wolever TMS, Tosh SM, Spruill SE, Jenkins AL, Ezatagha A, Duss R et al. Increasing oat β-glucan viscosity in a breakfast meal slows gastric emptying and reduces glycemic and insulinemic responses but has no effect on appetite, food intake, or plasma ghrelin and PYY responses in healthy humans: a randomized, placebo-controlled. Am J Clin Nutr 2020; 111. 10.1093/ajcn/nqz285. 10.1093/ajcn/nqz285 31828287

[22] Wolever TMS, Jenkins AL, Prudence K, Johnson J, Duss R, Chu Y et al. Effect of adding oat bran to instant oatmeal on glycaemic response in humans-a study to establish the minimum effective dose of oat β-glucan. Food Funct. 2018; 9. 10.1039/c7fo01768e. 10.1039/c7fo01768e 29480316

[23] Würsch P, Pi-Sunyer FX (1997). The role of viscous soluble fiber in the metabolic control of diabetes: a review with special emphasis on cereals rich in β-glucan. Diabetes Care.

[24] Horowitz M, Edelbroek MA, Wishart JM, Straathof JW (1993). Relationship between oral glucose tolerance and gastric emptying in normal healthy subjects. Diabetologia.

[25] Marathe CS, Rayner CK, Jones KL, Horowitz M (2013). Relationships between gastric emptying, postprandial glycemia, and incretin hormones. Diabetes Care.

[26] Cani PD, Joly E, Horsmans Y, Delzenne NM (2006). Oligofructose promotes satiety in healthy human: a pilot study. Eur J Clin Nutr.

[27] Cani PD, Lecourt E, Dewulf EM, Sohet FM, Pachikian BD, Naslain D (2009). Gut microbiota fermentation of prebiotics increases satietogenic and incretin gut peptide production with consequences for appetite sensation and glucose response after a meal. Am J Clin Nutr.

[28] Whelan K, Efthymiou L, Judd PA, Preedy VR, Taylor MA (2006). Appetite during consumption of enteral formula as a sole source of nutrition: the effect of supplementing pea-fibre and fructo-oligosaccharides. Br J Nutr.

[29] Chambers ES, Viardot A, Psichas A, Morrison DJ, Murphy KG, Zac-Varghese SEK (2015). Effects of targeted delivery of propionate to the human colon on appetite regulation, body weight maintenance and adiposity in overweight adults. Gut.

[30] Chambers ES, Morrison DJ, Frost G (2015). Control of appetite and energy intake by SCFA: what are the potential underlying mechanisms?. Proc Nutr Soc.

[31] Parnell JA, Reimer RA (2009). Weight loss during oligofructose supplementation is associated with decreased ghrelin and increased peptide YY in overweight and obese adults. Am J Clin Nutr.

[32] Calikoglu F, Barbaros U, Uzum AK, Tutuncu Y, Satman I (2021). The metabolic effects of pre-probiotic supplementation after Roux-en-Y gastric bypass (RYGB) surgery: a prospective, randomized controlled study. Obes Surg.

[33] Wölnerhanssen BK, Cajacob L, Keller N, Doody A, Rehfeld JF, Drewe J (2016). Gut hormone secretion, gastric emptying, and glycemic responses to erythritol and xylitol in lean and obese subjects. Am J Physiol Endocrinol Metab.

[34] Boets E, Gomand SV, Deroover L, Preston T, Vermeulen K, De Preter V (2017). Systemic availability and metabolism of colonic-derived short-chain fatty acids in healthy subjects: a stable isotope study. J Physiol.

[35] Verbeke K (2017). Quantification of plasma or serum short-chain fatty acids: choosing the correct blood tube. J Nutr Heal Food Sci.

[36] Scharrer E, Langhans W. Control of food intake by fatty acid oxidation. Am J Physiol. 1986; 250. 10.1152/AJPREGU.1986.250.6.R1003. 10.1152/ajpregu.1986.250.6.R1003 3717372

[37] Holst JJ (2007). The physiology of glucagon-like peptide 1. Physiol Rev.

[38] Xie C, Huang W, Sun Y, Xiang C, Trahair L, Jones KL (2023). Disparities in the glycemic and incretin responses to intraduodenal glucose infusion between healthy young men and women. J Clin Endocrinol Metab.

