# Supplementary material for: Effect of inulin on breath hydrogen, postprandial glycemia, gut hormone release, and appetite perception in RYGB patients: a prospective, randomized, cross-over pilot study
Source: Nutr Diabetes. 2024 Mar 6;14:9. doi: 10.1038/s41387-024-00267-5 (PMC10918168; doi:10.1038/s41387-024-00267-5)
Supplement: Supplementary file 1 — Supplemental Material [file 41387_2024_267_MOESM1_ESM.docx]

**Supplemental Table 1. Product characteristics**

|  | **Oligofructose enriched inulin**  (Orafti®Synergy1) | **Maltodextrin (MDX)** |
| --- | --- | --- |
| **Total** | 28,7 g | 15,5 g |
| **Energy (kcal)*** | 61.4 kcal | 60,1 |
| **Fat** | 0 g | 0,0 |
| **CHO** | 2,9 | 15,5 |
| **Protein** | 0 g | 0,0 |
| **Inulin** | 25 g | 0,0 |

Values for fat, carbohydrate (CHO) and protein were provided by the supplier. *Energy content is based on the energy conversion factor of 8 kJ/g or 2 kcal/g as laid down for all fibres in the EU


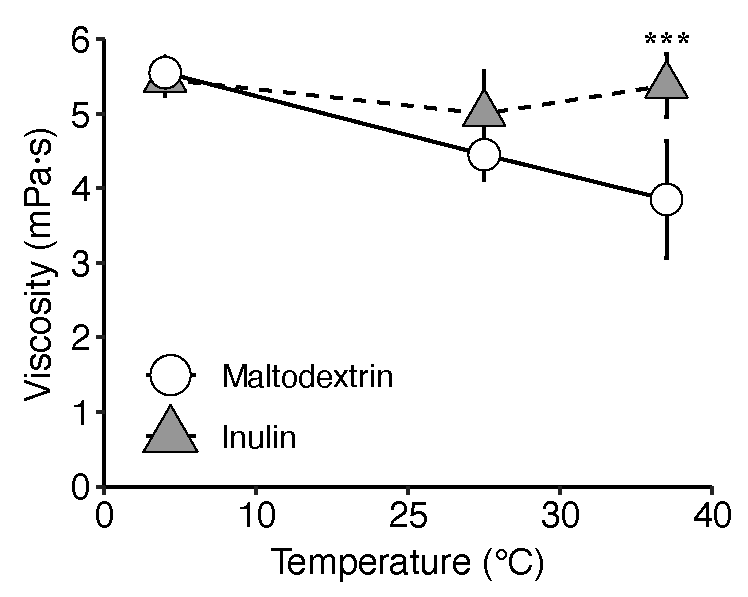


**Supplemental Figure 1.** Viscosity as a function of temperature for (a) maltodextrin (MDX) and (b) inulin dissolved in 300 ml orange juice
